# PGC-1β cooperating with FOXA2 inhibits proliferation and migration of breast cancer cells

**DOI:** 10.1186/s12935-019-0810-5

**Published:** 2019-04-11

**Authors:** Jia Cao, Xi Wang, Danni Wang, Rong Ma, Xiaohan Li, Huimin Feng, Jia Wang, Shihai Liu, Libin Wang

**Affiliations:** 1 School of Clinical Medicine, Ningxia Medical University, Yinchuan, 750004 China; 2 Beijing National Biochip Research Center Sub-Center in Ningxia, The General Hospital of Ningxia Medical University, Yinchuan, 750004 China; 3 Medical Animal Lab, The Affiliated Hospital of Qingdao University, Qingdao, 266003 China

**Keywords:** PGC-1β, FOXA2, Breast cancer, Proliferation, Migration

## Abstract

**Background:**

Breast cancer is one of the most common malignancy among females from the worldwide cancer incidence statistics. Peroxisome gamma coactivator-1β (PGC-1β) has long been identified to be involved in this type of tumorigenesis. However, the mechanisms of PGC-1β in human breast cancer have not been fully understood and the function requires to be further elucidated.

**Methods:**

mRNA and protein expression of PGC-1β and FOXA2 in breast cancer tissues and cell lines were determined by qRT-PCR and Western Blotting, respectively. To further visualize the expression and localization of PGC-1β and FOXA2, immunochemistry and immunofluorescence staining methods were employed. The effect of PGC-1β and FOXA2 on cell proliferation and migration were evaluated by CCK8, clone formation, transwell and wound-healing assays, which has been done either with stable PGC-1β knockdown or FOXA2 overexpression in vitro. Xenografts model of nude mice were used to evaluate tumor growth in vivo. In addition, proteins expression of the PI3K-AKT-mTOR signaling pathway involved in the regulation of breast cancer were detected by Western Blotting.

**Results:**

Our results showed that PGC-1β was upregulated and FOXA2 was downregulated in breast cancer tissues and cell lines. These two proteins can be interacted with each other to form the complex. Also, we found the combination of PGC-1β interference with FOXA2 overexpression significantly inhibited cell proliferation and migration in vitro as well as tumor growth in vivo. We further identified that PGC-1β and FOXA2 strongly correlated with the PI3K-AKT-mTOR signaling pathway, and they exerted their biological functions by activating this pathway.

**Conclusions:**

We demonstrated that downregulation of PGC-1β combined with overexpression of FOXA2 obviously inhibited the function of breast cancer cells through regulating the PI3K-AKT-mTOR pathway.

## Background

Breast cancer is one of the most common malignancy among women all over the world [1]. The latest survey shows more than 2000 million newly diagnosed patients of breast cancer have occurred in the worldwide. And the incidence and mortality of breast cancer have increased by nearly 18% since 2008 [2]. Breast cancer is a diverse hormone-dependent carcinoma, with both genetic and epigenetic changes leading to initiation, progression and metastasis [3]. Despite significant advances have been made in the biological development of breast cancer, the occurrence of metastasis still leads to poor prognosis and low survival. Therefore, discovery of the new biomarkers and development of the targeted therapeutics strategies are urgently needed for diagnosis and treatment of breast cancer.

The peroxisome proliferator-activated receptor (PPAR) gamma coactivator-1 (PGC-1) family of transcriptional coactivators, mainly including PGC-1α and PGC-1β, have emerged as being major regulators of mitochondrial biogenesis and cellular metabolism to regulate the cellular bioenergetics [4, 5]. The members of PGC-1 family are multifunctional transcriptional co-regulators that take on “molecular switches” in many physiological and pathological processes [6]. They are highly expressed in oxidative capacity and energy demanding tissues, such as heart, brain, skeletal muscle and brown adipose tissue [7–10] and considered to be essential for many of exercise response in skeletal muscle [11]. Specifically, PGC-1α and PGC-1β can interact with each other, or interact with multiple transcription factors, such as forkhead/winged helix protein family member FOXO1, and nuclear respiratory factor-1/2 (NRF1/2) in tissue specific manner [5, 12] to modulate in mitochondrial biogenesis, fatty acid β-oxidation, adipogenesis, adaptive thermogenesis and tricarboxylic acid cycle [13, 14].

The PGC-1β protein, encoded by the PPARGC1β, is located on chromosome 5q33.1. PGC-1β has involved in many biological processes. For example, it is a biological candidate gene in relation to obesity and obesity related type 2 diabetes [15]; hepatic PGC-1β as a transcriptional gatekeeper of mitochondrial function, contributes to hepatocellular carcinoma progression [16]. It has been reported that PGC-1β was closely related to the tumor biological properties and cancer cells proliferation via metabolic and redox pathways [17]. Our previous research proved that PGC-1β was significantly overexpressed in breast cancer and inhibited the apoptosis of breast cancer cells via mTOR signaling pathway [18]. Therefore, we postulated the PGC-1β may play an important role in the development and progression of breast cancer.

The forkhead box protein A2 (FOXA2), a pioneer transcription factor, is one of the members of forkhead/winged-helix family that comprises the transcription factors FOXA1, FOXA2 and FOXA3 in mammals [19–21]. Previous studies have shown that FOXA2 is a critical regulator of embryonic development, and plays a key role in the pathogenesis and occurrence of various cancers, such as prostate cancer [22, 23], liver cancer [24, 25], pancreatic cancer [26], and breast cancer [27, 28]. The expression of FOXA2 can prevent the development and progression of the above cancers [29]. In addition, FOXA2 can regulate transcription factor expression, and involve in a variety of biological processes, including cell growth, proliferation, migration, differentiation and autophagy in multiple human tumors [30–33]. Given that FOXA2 exerts essential roles in these processes, the loss or gain of FOXA2 function can modify the tumor biological behavior and participate in tumorigenesis.

PGC-1β and FOXA2 have some similar functions, such as regulating the lipid metabolism and modulating the function of other transcription factors. In the present study, we aimed to elucidate the role of PGC-1β and FOXA2 in breast cancer progression. We found that PGC-1β is upregulated and FOXA2 is downregulated in breast cancer tissues and cell lines. PGC-1β cooperating with FOXA2 can regulate the tumor cells proliferation, migration and apoptosis in vitro and vivo. Meanwhile, we identified that PGC-1β interacts with FOXA2, and it synergizes with FOXA2 to inhibit the biological functions of breast cancer cells through regulating the PI3K-AKT-mTOR signaling pathway. Together, our study highlights the PGC-1β and FOXA2 may be potential targets for the development of breast cancer.

## Materials and methods

### Cell line and cell culture

Human breast cancer cell lines (MCF-7 and MDA-MB-231) and normal human breast cell line (MCF-10A) were purchased from the Cell bank of the Chinese Academy of Sciences/American Type Culture Collection (ATCC, VA, USA). Human breast cancer cell line (BT-549) was donated by Dr. Liu from the Affiliated Hospital of Qingdao University. The above cells were maintained and cultured in DMEM HIGH GLUCOSE medium (HyClone, Logan, USA) supplemented with 10% fetal bovine serum (Gibco, Australia) and 1% penicillin–streptomycin solution in the incubator at 37 °C under 5% CO_2_.

### Clinical cancer tissue samples

A total of 30 paired breast cancer tissues and adjacent non-tumor tissues samples were collected and obtained undergoing breast carcinectomy from the General Hospital of Ningxia Medical University. The fresh samples were frozen and stored in liquid nitrogen until further use. All the tissue samples were signed by patients with informed consent.

### Western Blotting

The cells were harvested and lysed using RIPA lysis buffer containing configure RIPA, PIC, and PMSF at a ratio of 100:2:1 (Beyotime Biotechnology, Shanghai, China). The total protein was extracted and the protein concentration was detected by BCA protein reagent kit (Thermofisher Scientific, Inc). The samples were electrophoresed on 10% SDS-PAGE and transferred to PVDF membranes. The membranes were blocked with 5% defatted milk in TBST for 1 h at room temperature and then incubated with specific primary antibodies overnight at 4 °C (Table 1). Subsequently, the HRP-conjugated secondary antibodies were incubated at room temperature for 1.5 h. The protein bands were detected and scanned by BioImaging Systems (BIO-RAD, CA, USA). Anti-GAPDH antibody was used as an internal control to normalize protein levels.

**Table 1:** The antibodies used in the western blotting analysis

Protein	Producer	Catalog number	Dilution
PGC-1β	NOVUS	NB110-58858G	1:500
FOXA2	Abcam	ab108422	1:1000
GAPDH	Abcam	ab128915	1:5000
p-AKT	Abcam	ab38449	1:500
AKT	CST	#9272	1:1000
PI3K	CST	#4257	1:500
mTOR	CST	#2983	1:500
Rictor	CST	#2114	1:1000
Raptor	CST	#2280	1:1000
PDK1	CST	#3062	1:1000

*CST* cell signaling technology

### Real time quantitative reverse transcription-PCR (qRT-PCR)

Total RNA was extracted from tissues and cells using TRIzol reagent (Invitrogen, CA, USA) as per manufacturer’s instructions. cDNA synthesis was performed by using a reverse transcription kit (TaKaRa, ShangHai, China) according to the manufacturer’s protocols. The PCR amplification was performed with specific primers and carried out using the SYBR-Green PCR system (Takara Bio, Inc). GAPDH or β-actin served as internal control. Calculation of the relative expression of each gene was quantified by the 2^−ΔΔCt^ method.

### Plasmids and cell transient transfection

The plasmids, including shRNA for PGC-1β (sh-PGC-1β), shRNA-NC (NC), pcDNA3.1-PGC-1β (PGC-1β), pcDNA3.1-FOXA2 (FOXA2), shRNA for FOXA2 (sh-FOXA2) were designed and synthesized by Shanghai Genechem Co.Ltd (Shanghai, China). MCF-7 and MDA-MB-231 cells were plated in six-well plates, and the cells were transfected with 2.5 µg plasmid using Lipofectamine 3000 (Invitrogen, CA, USA) when the cell density reaches 70–80% confluence according to the manufacturer’s protocol. Cell transfection efficiency was observed under fluorescence microscope and examined using qRT-PCR and western blot assays.

### Cell proliferation and colony formation assays

The cell proliferation ability was assessed with CCK-8 assay and clone formation assay. For the CCK-8 assay, MCF-7 or MDA-MB-231 cells were seeded in 96-well plates and were cultured for 24, 48, 72 h, respectively. The cells were then treated with CCK-8 reagent (KeyGEN BioTECH, Jiangsu, China) and further cultured for 2 h according to the manufacturer’s instructions. The optical density was measured using the spectrophotometer (Glomax Multi Detection Systerm, Promega, USA) at 450 nm. Each group of experiments included five replicates and repeated three times.

For the colony formation assay, 8 × 10^3^ cells were added into 6-well plate and suspended in DMEM medium. The cells were exchanged every 3 days until the 12 days. Then, these dishes were fixed with 100% methanol for 15 min and stained with crystal violet for 10 min. The number of colonies were observed and calculated from representative areas. All experiments were performed in triplicate.

### Migration assays

The migration ability of the tumor cells were examined by transwell and wound healing assays. The cells were seeded in twelve-well plate and transfected with interfering or overexpressing plasmids for 48 h. The cell monolayer was wounded by scraping with 200 µl pipet tip and the exfoliated cells were washed with PBS. Subsequently, the speed of wound closure was observed and photographed under a microscope. Cell mobility was assessed by measuring three randomly perpendicular wound width. For the transwell assay, Corning Incorporated transwell Chambers (Corning, 8 μm, NY, USA) were used to detect the cells of migration capacity. The chambers were placed into a 24-well plate containing culture medium supplemented with 20% FBS as a chemo-attractant. After transfection with 48 h, MCF-7 and MDA-MB-231 cells were collected and suspended in serum-free medium and loaded onto the upper chamber, incubated and allowed to migrate at for 24 h and 48 h, respectively. The cells were fixed with 4% paraformaldehyde for 15 min and stained with crystal violet for 10 min. The non-invading cells were removed using swab, whereas total numbers of cells were imaged and counted from representative areas under a microscope (Olympus, Tokyo, Japan).

### Flow cytometry

MCF-7 and MDA-MB-231 cells were planted in six-well plate and treated with transfection reagent for 48 h, and the cells were digested with trypsin without EDTA and counted 1 × 10^6^ cells for analysis of each sample. After being washed twice with cold PBS, the cells were collected and prepared to form a single cell suspension in a binding buffer, and stained with AnnexinV-APC for 10 min and propidium iodide (PI) for 10 min according to instructions of the AnnexinV-APC/PI apoptosis detection kit (BestBio, Shanghai, China). Samples were examined using BD flow cytometer and the data were analyzed with FlowJo software (TreeStar Corporation, Ashland, OR, USA).

### Immunofluorescence staining

Cells were grown in 24-well chamber slides (NEST, USA) for 48 h, and washed with cold PBS and fixed in 4% paraformaldehyde for 15 min. Subsequently, the coverslips were permeabilized with 0.3% Triton X-100 for 6 min and blocked with 5% normal goat serum for 30 min at room temperature. Then, the cells were incubated with anti-PGC-1β (ab176328, Abcam) and anti-FOXA2 (ab117542, Abcam) overnight at 4 °C, and followed by incubation with fluorescent secondary antibody for 1 h at room temperature. The nucleus was visualized with DAPI solution (dilution: 1:1000, Sigma, USA) for 6 min in the dark. Confocal laser scanning microscope was utilized for image analysis.

### Immunohistochemistry

The paraffin-embedded tissues were deparaffinized using xylene and rehydrated through graded concentrations of alcohol solution. The slides were incubated with 0.3% hydrogen peroxide for 30 min to inactivate endogenous peroxidase prior to the infiltration of the citrate buffer with the tissue parts to repair the antigen in a microwave. The slides were rinsed with PBS for 15 min, blocked with 5% goat serum for 1 h at room temperature and incubated with the primary antibodies at 4 °C overnight. Subsequently, the slides were incubated with biotinylated secondary antibody at room temperature for 1 h. The sections were visualized with 3, 3′-diaminobenzidine (DAB) chromogenic fluid and counterstained with hematoxylin (CWBIO, Beijing, China).

### Co-immunoprecipitation assay

293T and MCF7 cells were seeded in 10 cm dish, and were co-transfected with PGC-1β and FOXA2 overexpression plasmids by Lipofectamine 3000. The cells were lysed using RIPA lysis buffer containing protease inhibitor cocktails (Thermo, USA). The cell lysates were used in immunoprecipitation after centrifuged at 12,000 rpm for 20 min. The proteins extracted from the cells were incubated the primary antibody or normal mouse/rabbit IgG at 4 °C for 4–6 h and then incubated with 30 μl Protein A/G Plus-Agarose beads (GE Healthcare Bio-Sciences, Sweden) for overnight. The immune complexes were precipitated by centrifuged for 5 min, and the precipitates were washed three times with corresponding washing buffer. The precipitates were resuspended in 5× SDS-PAGE sample loading buffer to boiled (100 °C) for 10 min for western blot assay.

### Tumor xenograft model

MDA-MB-231 cells were infected with lentivirus vector and subcutaneously implanted into 4–6-week-old female BalB/C nude mice, which purchased from the Beijing Vital River Laboratory Animal Technology Co. Ltd (Beijing, China). The tumor nodules were assessed with electronic calipers by recording tumor length and width for 15 days. Subsequently, the mice were euthanized and tumor mass were collected, weighed and photographed. All animal experiments were performed in accordance with principles and guidelines approved by the Ethics Committee of Animal Research.

### Statistical analysis

Statistical testing was performed with the SPSS 17.0, GraphPad Prism version 6.0 and Microsoft Office Excel 2013 software. All assays were conducted three times and results were presented as mean ± SD. Statistical differences among groups were determined by two-tailed Student t test or one-way analysis of variance (one-way ANOVA). Results were considered significant when P values < 0.05.

## Results

### Expression levels of PGC-1β and FOXA2 in breast cancer tissues and cells

**Fig. 1:**
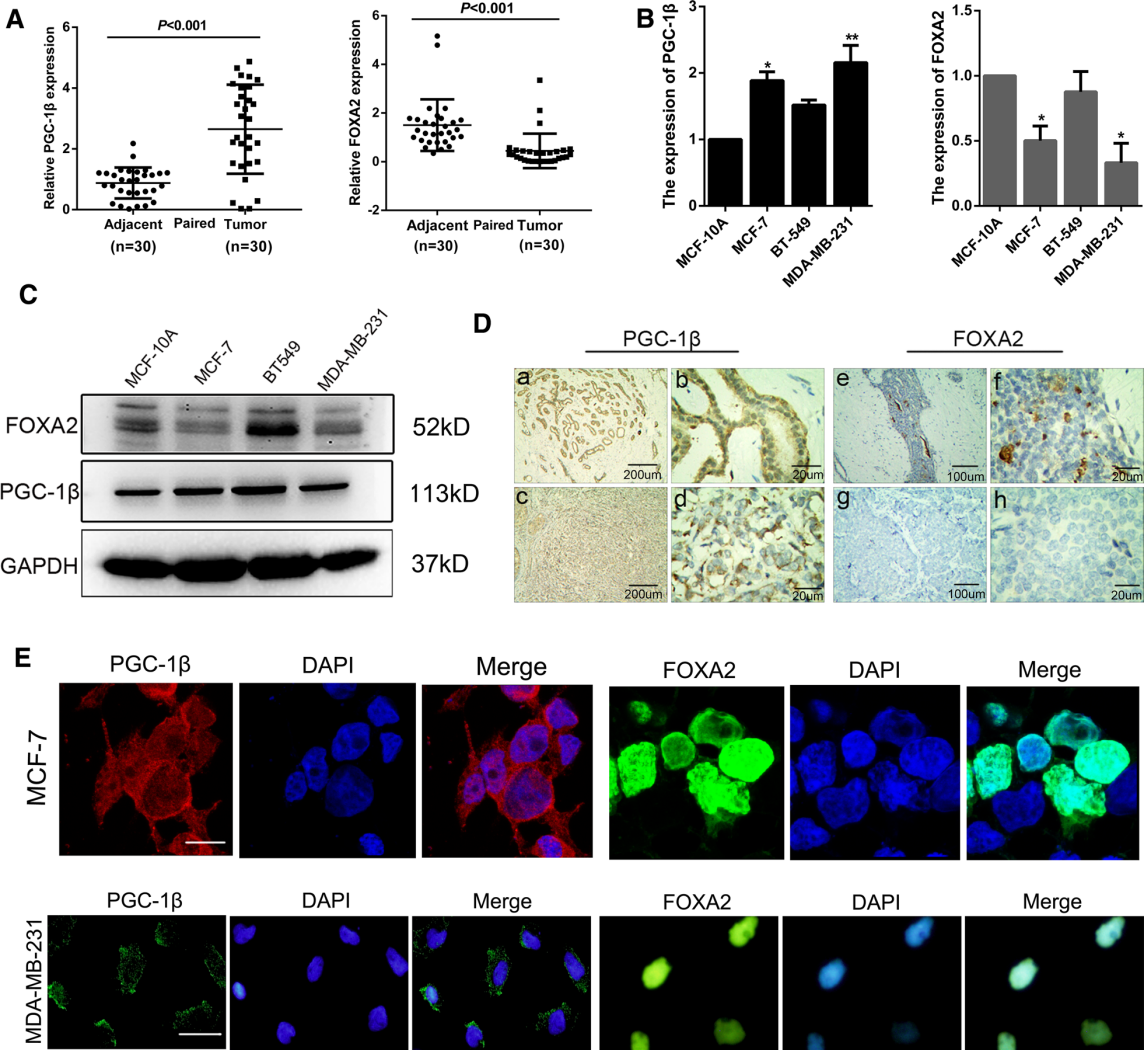
The expression levels of PGC-1β and FOXA2 in breast cancer tissues and cells. **a** The expression levels of PGC-1β and FOXA2 in breast cancer tissues and matched adjacent tissues by qRT-PCR. **b**, **c** The expression levels of PGC-1β and FOXA2 mRNA and proteins were detected by qRT-PCR and western blot. **d** Representative pictures of PGC-1β and FOXA2 expression by immumohistochemical staining in breast cancer and non-cancer tissues (PGC-1β expression in non-cancer tissues: a ×40, b ×400; PGC-1β expression in breast cancer tissues: c ×40, d ×400; FOXA2 expression in non-cancer tissues: e ×100, f ×400; FOXA2 expression in breast cancer tissues: g ×100, h ×400). **e** Subcellular localization of PGC-1β and FOXA2 were detected by immunofluorescence staining. (MCF-7: original magnification ×630, scale bar = 10 μm; MDA-MB-231: original magnification ×400, scale bar = 20 μm). All values were expressed as mean ± SD. *P < 0.05, **P < 0.01

To detect expression of PGC-1β and FOXA2 in breast cancer, qRT-PCR was performed on 30 pairs of breast cancer tissues and adjacent normal tissues. Elevated levels of PGC-1β and low levels of FOXA2 were observed in breast cancer tissues (Fig. 1a). Moreover, we examined the expression of both genes in human breast cancer cell lines (MCF-7, MDA-MB-231 and BT-549) and normal breast cell line (MCF-10A). Consistent with the findings in the clinical breast cancer specimens, the PGC-1β mRNA and protein expression levels were significantly upregulated in the breast cancer cell lines. Whereas, the expression of FOXA2 was negatively correlated with PGC-1β (Fig. 1b, c). Results from human breast cancer tissues indicated that abnormal expression of PGC-1β was closely related to the clinicopathological characteristics of patients, such as Lymph node metastasis (P = 0.026) and Progesterone receptor (P = 0.026) (Table 2). Immunohistochemical assay showed strong staining of PGC-1β and weak staining of FOXA2 were observed in breast tumor tissues, which was consistent with the above results (Fig. 1d). Moreover, we detected the localization of PGC-1β and FOXA2 through immunofluorescence assay. It was found that PGC-1β localized mainly in the cytoplasm, and FOXA2 localized mostly in the nucleus (Fig. 1e). Collectively, these data clearly demonstrated that abnormal expression of PGC-1β and FOXA2 were frequent event in breast cancer, and they might play vital roles in the development and progression of breast cancer.

**Table 2:** The relevance of PGC-1β expression to clinicopathological features of breast cancer patients

Characteristics	N (%)	PGC-1β expression		P value
		Positive (%)	Negative (%)	
Age (years)				0.563
< 50	11 (36.7)	7 (63.6)	4 (36.4)	
≥ 50	19 (63.3)	14 (73.7)	5 (26.3)	
TNM stage				0.207
I–II	22 (73.3)	14 (63.6)	8 (36.4)	
III	8 (26.7)	7 (87.5)	1 (12.5)	
Tumor size (cm)				0.469
≤ 2	13 (43.3)	10 (76.9)	3 (23.1)	
> 2	17 (56.7)	11 (64.7)	6 (35.3)	
Lymph node metastasis				0.026^*^
Negative	11 (36.7)	5 (45.5)	6 (54.5)	
Positive	19 (63.3)	16 (84.2)	3 (15.8)	
Estrogen receptor				0.207
Negative	8 (26.7)	7 (87.5)	1 (12.5)	
Positive	22 (73.3)	14 (63.6)	8 (36.4)	
Progesterone receptor				0.026^*^
Negative	11 (36.7)	5 (45.5)	6 (54.5)	
Positive	19 (63.3)	16 (84.2)	3 (15.8)	
HER2 receptor				0.232
Negative	15 (50.0)	9 (60.0)	6 (40.0)	
Positive	15 (50.0)	12 (80.0)	3 (20.0)	

*P* value from Chi square test (^*^P < 0.05)

### PGC-1β cooperating with transcription factor FOXA2

**Fig. 2:**
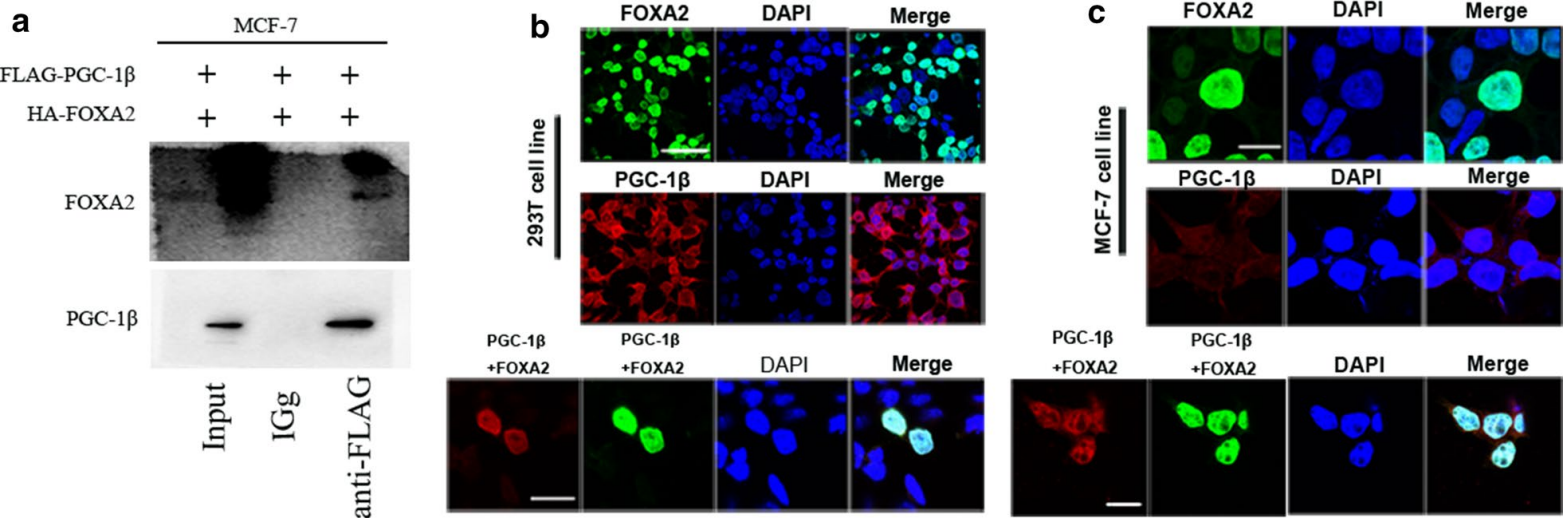
PGC-1β cooperating to transcription factor FOXA2. **a** Combination of PGC-1β and FOXA2 proteins. MCF7 cells were transfected with FLAG-PGC-1β and HA-FOXA2. Input group was used as a positive control, and normal IgG group was used as a negative control. **b**, **c** Colocalization of PGC-1β and FOXA2 in 293T and MCF-7 cell lines by immunofluorescence staining (magnification ×400; scale bar = 20 μm)

To confirm the interaction of PGC-1β and FOXA2, we detected the binding of two proteins in MCF-7 cell by Co-immunoprecipitation (Co-IP) assay. The FLAG-tagged PGC-1β and HA-tagged FOXA2 were co-expressed into MCF-7 cells. As shown in Fig. 2a, anti-FLAG antibody was co-precipitated with HA-FOXA2. However, HA-FOXA2 did not pull down FLAG-PGC-1β. Co-IP assay indicated that endogenous PGC-1β protein might target to FOXA2 in breast cancer. The subcellular colocalization of PGC-1β and FOXA2 were examined to further determine whether PGC-1β can combine with FOXA2 physically through immunofluorescence (IF) staining. The overexpression plasmids of PGC-1β and FOXA2 were transfected into HEK293T and MCF-7 cell lines. The cells were fixed and incubated with anti-PGC-1β and anti-FOXA2 antibodies. PGC-1β was found to be mainly expressed in the cytoplasm in the cells transfected with PGC-1β plasmid alone, and FOXA2 was expressed in the nucleus in the cells transfected with FOXA2 plasmid only. Interestingly, PGC-1β and FOXA2 were co-localized in the nucleus when the cells were co-transfected with PGC-1β and FOXA2 overexpression plasmids (Fig. 2b, c). These findings suggested that PGC-1β and FOXA2 can physically interact with each other and form complex.

### Transient PGC-1β-suppressed and FOXA2-overexpressed cells were constructed

**Fig. 3:**
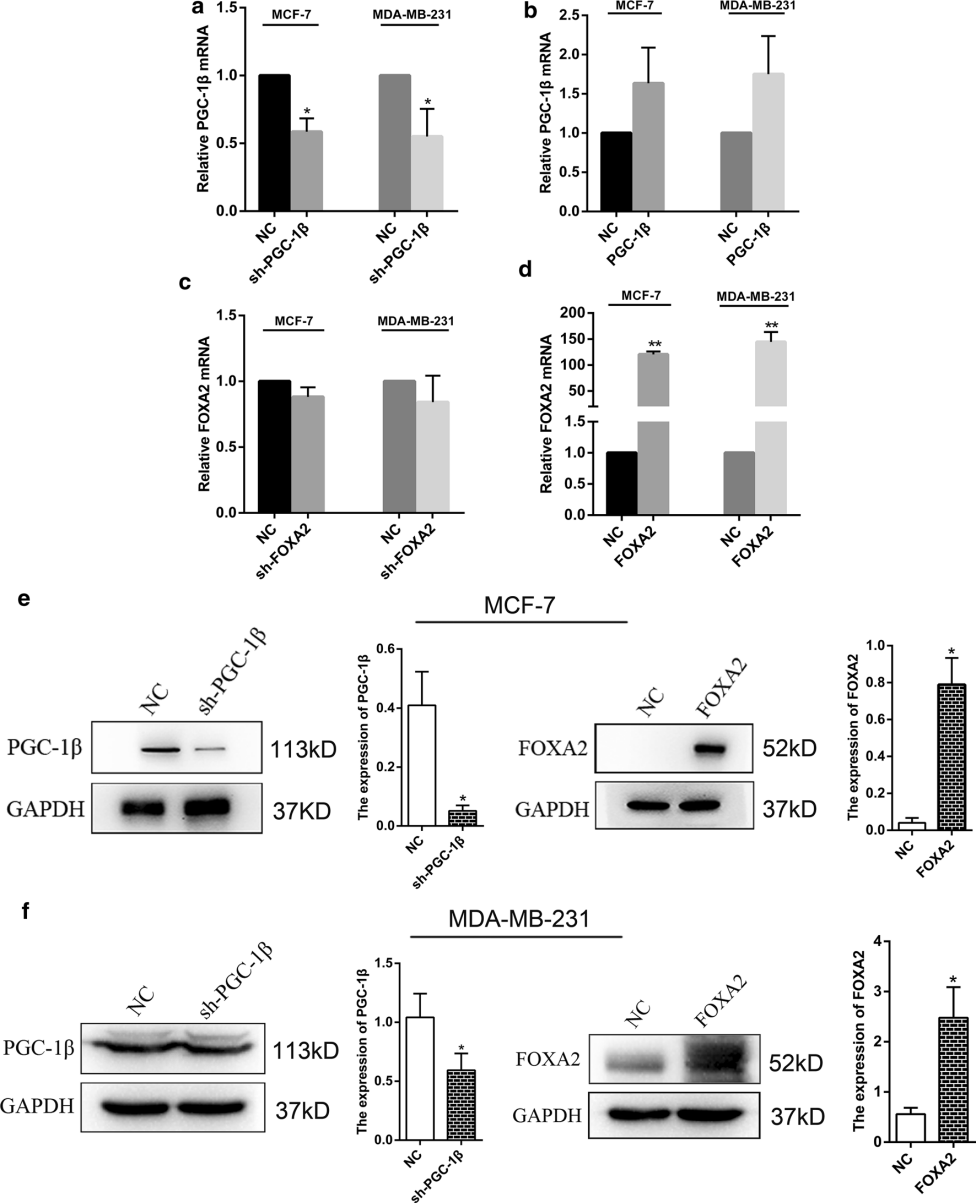
The transfection efficiency of PGC-1β and FOXA2 were detected in MCF-7 and MDA-MB-231 cells. **a**, **b** The transfection of PGC-1β interference and overexpression vectors were detected in MCF-7 and MDA-MB-231 cell lines by qRT-PCR. **c**, **d** The transfection of FOXA2 interference and overexpression vectors were detected in MCF-7 and MDA-MB-231 cell lines by qRT-PCR. **e**, **f** The protein expression levels of PGC-1β and FOXA2 were detected by western blot. All bar graphs depicted quantification of triplicate results with mean ± SD. *P < 0.05, **P < 0.01

The specific shRNA against PGC-1β (sh-PGC-1β) and overexpression against FOXA2 (FOXA2) were transfected with MCF-7 and MDA-MB-231 cells. The results indicated that compared with the corresponding control group (NC), transfection of sh-PGC-1β reduced the expression of endogenous PGC-1β (Fig. 3a) and transfection of FOXA2 overexpression significantly increased FOXA2 expression (Fig. 3d) by qRT-PCR assay. At the same time, the MCF-7 and MDA-MB-231 cells were also transfected with the overexpression plasmid of PGC-1β and the interference plasmid of FOXA2. The results showed that there were no significant difference between the two groups (*P *> 0.05, Fig. 3b, c). Western Blotting results showed that transfected with PGC-1β interference and FOXA2 overexpression vector, the proteins expression of PGC-1β and FOXA2 were decreased and increased, respectively (Fig. 3e, f). Therefore, the PGC-1β interference and FOXA2 overexpression vectors were used for the subsequent experimental studies.

### Knockdown of PGC-1β combined with overexpression of FOXA2 inhibited proliferation and migration of breast cancer cells in vitro

**Fig. 4:**
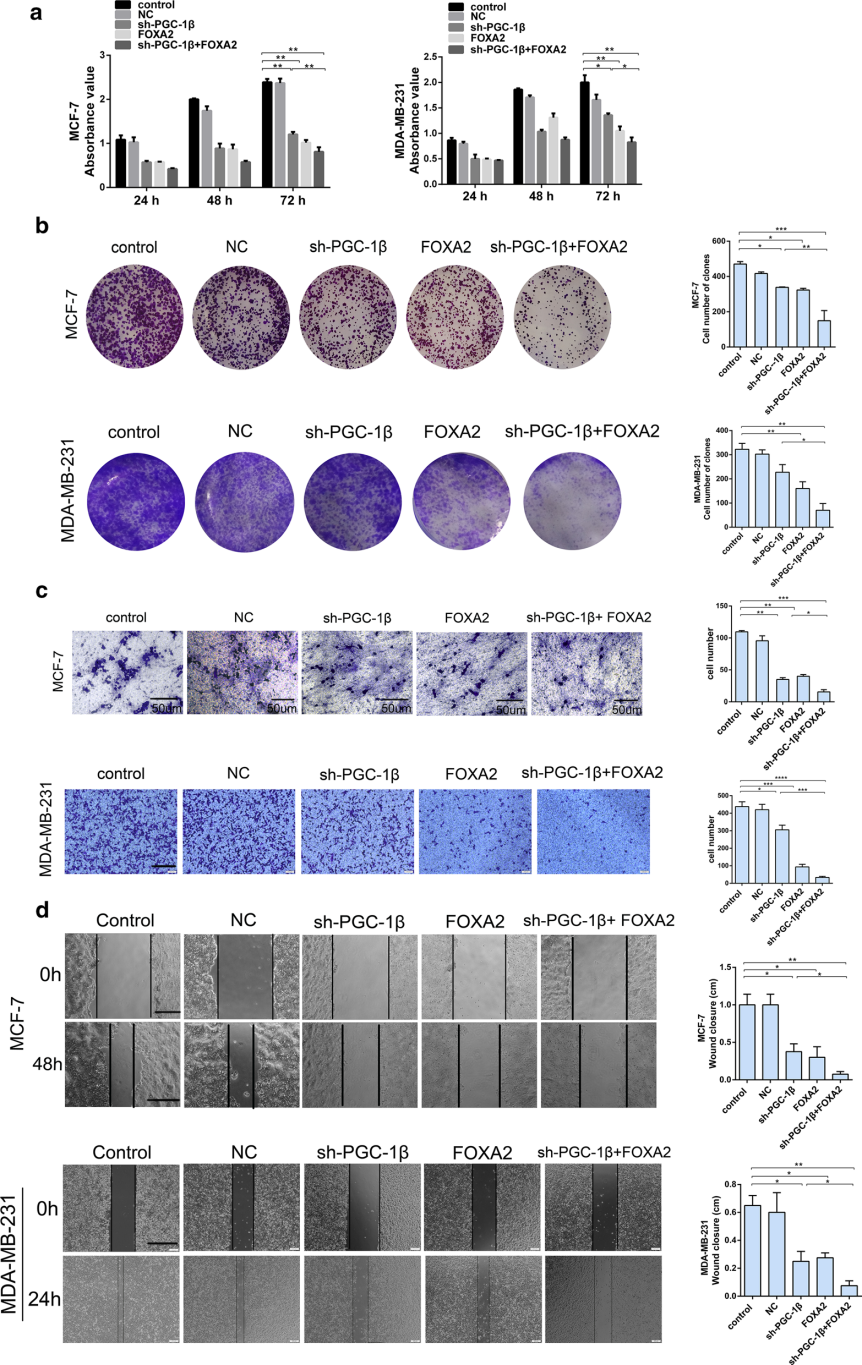
Knockdown of PGC-1β combined with overexpression of FOXA2 inhibit proliferation and migration of breast cancer cells. MCF-7 and MDA-MB-231 cells were transfected with overexpression plasmid (FOXA2) and interference plasmid (sh-PGC-1β) to detect the cell proliferation and migration. **a** Cell growth was evaluated by CCK-8 assay. **b** In vitro proliferative ability of two cell lines were detected by clone formation assay. **c** Cell metastatic was assessed using Transwell assay. (MCF-7: original magnification ×200, scale bar = 50 μm; MDA-MB-231: original magnification ×40, scale bar = 200 μm). **d** In vitro migration ability were detected using scratch wound healing assay (scale bar = 200 μm). *P < 0.05, **P < 0.01. ***P < 0.001

To explore the biological functions of PGC-1β and FOXA2 in breast cancer progression, we examined the effects of both genes on cell proliferation and clonogenicity by CCK-8 and colony formation assays. The results demonstrated that the cell viability and proliferation were inhibited in MCF-7 and MDA-MB-231 cells after knockdown of PGC-1β or overexpression of FOXA2. It is worth noting that co-transfection with plasmids of sh-PGC-1β and FOXA2 dramatically reduced cell proliferation ability compared with individual transfection (Fig. 4a, b). Afterwards, we assessed whether PGC-1β and FOXA2 were involved in regulating the cells migration. Consistent with the above results, the cell migration was more significantly inhibited when co-transfected with both of plasmids (Fig. 4c, d). Overall, knockdown of PGC-1β combined with overexpression of FOXA2 apparently inhibited proliferation and migration of breast cancer cells in vitro.

### Suppression of PGC-1β in combination with overexpression of FOXA2 inhibited xenograft tumor growth and promoted apoptosis of breast cancer

**Fig. 5:**
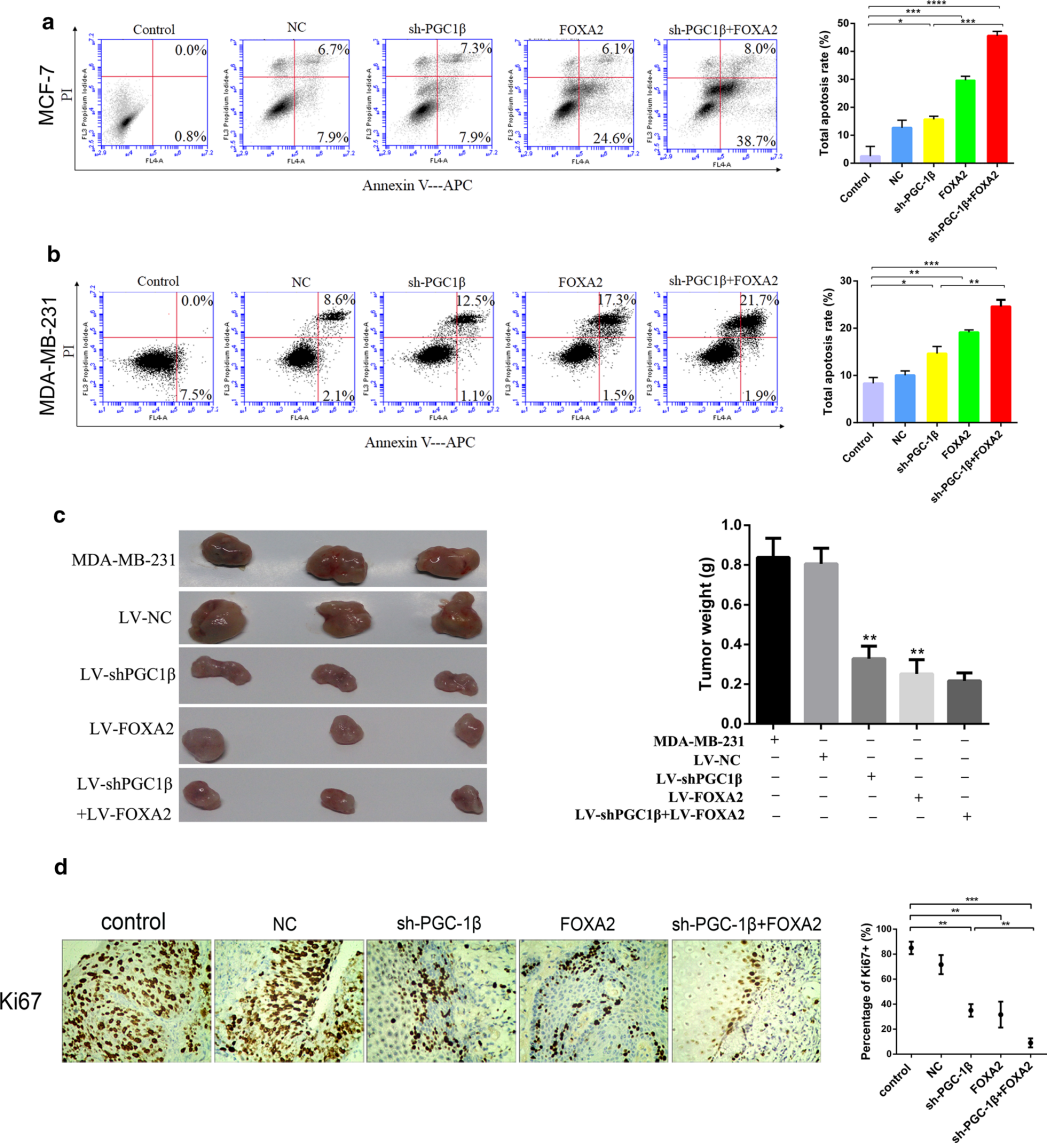
Downregulation of PGC-1β combined with FOXA2 promote cell apoptosis and suppress xenograft tumor growth. **a**, **b** Apoptosis assay was performed by flow cytometry using the Annexin V/PI staining. **c** Cells from each treatment group were injected subcutaneously into nude mice. The xenograft models were performed to detect the effect of PGC-1β and FOXA2 on breast cancer growth in vivo according to the tumor weight. **d** The expression of Ki67 were detected by immunohistochemical assay. *P < 0.05, **P < 0.01, ***P < 0.001

With AnnexinV-APC/PI staining and flow cytometry analysis, we evaluated the effects of PGC-1β and FOXA2 on cell apoptosis. The results indicated that downregulation of PGC-1β or upregulation of FOXA2 promoted apoptosis of MCF-7 and MDA-MB-231 cells compared to the control groups. Of note, co-transfection with plasmids of sh-PGC-1β and FOXA2 significantly increased cell apoptosis in comparison to that in cells with individual transfection (Fig. 5a, b). Subsequently, mouse subcutaneous xenograft experiments were performed to detect the effects of PGC-1β and FOXA2 on tumor growth in vivo. The tumor weight of xenografts derived from LV-sh-PGC-1β or LV-FOXA2 were significantly lighter compared with LV-NC. However, there was no significant difference in tumor weight when co-transplanted with LV-sh-PGC-1β and LV-FOXA2 (P > 0.05, Fig. 5c). Additionally, immunohistochemistry assay proved that PGC-1β inhibition or FOXA2 overexpression effectively reduced Ki67 expression compared with the NC group (Fig. 5d). The above results indicated that the combination of sh-PGC-1β and FOXA2 further suppressed xenograft tumor growth and promoted apoptosis in breast cancer.

### The combination of PGC-1β and FOXA2 inhibited breast tumor growth and metastasis through regulating the PI3K-AKT-mTOR signaling pathway

**Fig. 6:**
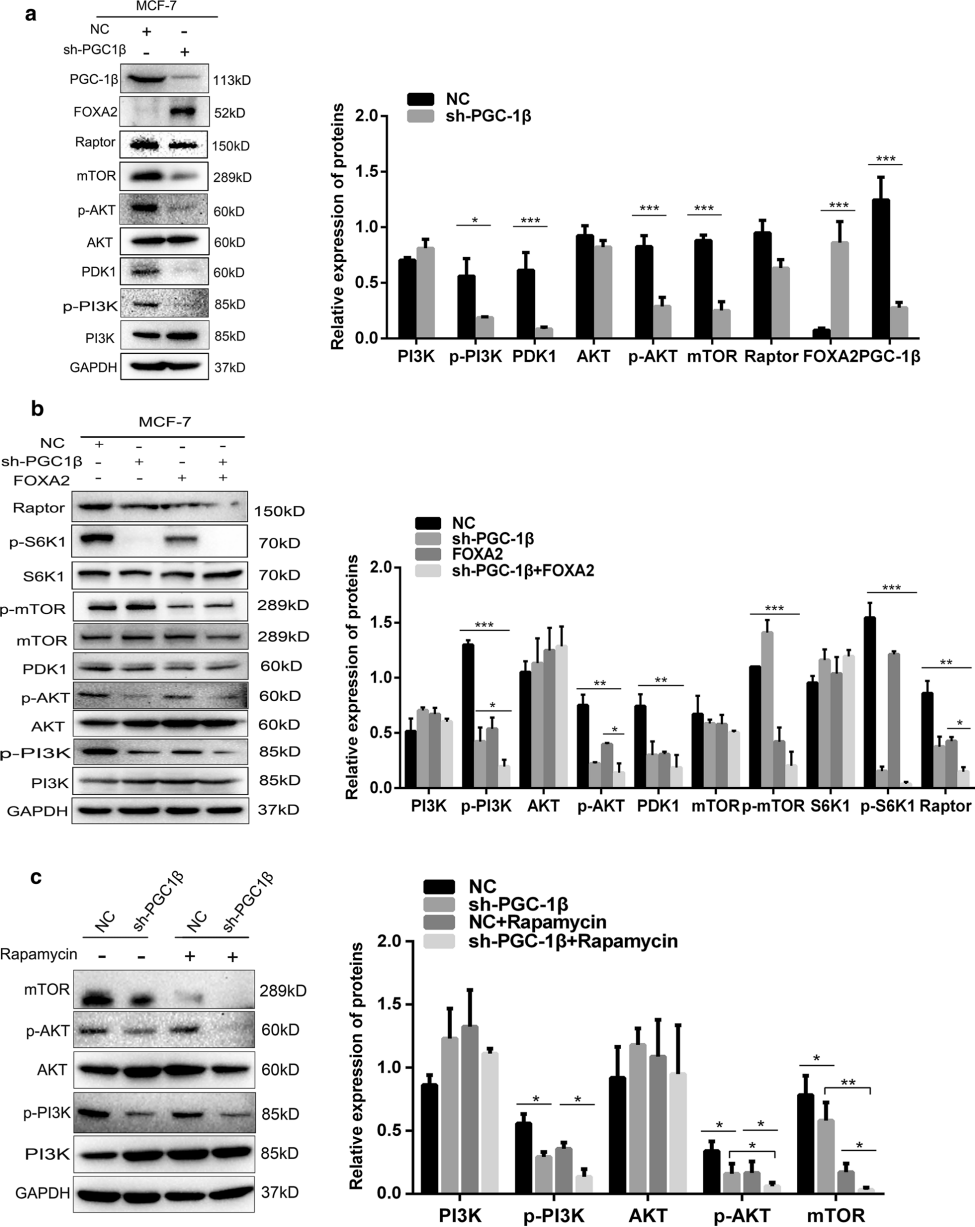
The combination of PGC-1β and FOXA2 inhibits breast tumor growth and metastasis through regulating the PI3K-AKT-mTOR signaling pathway. **a** The related proteins of PI3K-AKT-mTOR signal pathway were examined by western blot. GAPDH was used as loading control. **b** The related proteins of PI3K-AKT-mTOR signal pathway were evaluated in MCF-7 cells when co-transfected with sh-PGC-1β and FOXA2. **c** The protein levels of PI3K, p-PI3K, AKT, p-AKT, mTOR were detected after treatment of mTOR inhibitor rapamycin (1000 nM). Data are shown as mean ± SD from three independent experiments. *P < 0.05, **P < 0.01, ***P < 0.001

PI3K-AKT-mTOR pathway has been reported that frequently involves in variety of human cancers and regulates numerous cellular processes, such as cell growth, proliferation and migration [34]. To investigate the molecular mechanisms of PGC-1β and FOXA2 in breast cancer progression, we detected the expression of related proteins of PI3K-AKT-mTOR pathway. The results showed that the expression of PGC-1β, p-PI3K, p-AKT, PDK1, mTOR and Raptor were downregulated in MCF-7 cells when transfected with sh-PGC-1β. However, the expression of FOXA2 was increased (Fig. 6a). Then, we found that the expression of p-PI3K, p-AKT, PDK1, p-mTOR, p-S6K1 and Raptor were significantly decreased when co-transfected with sh-PGC-1β and FOXA2 (Fig. 6b). To further prove the role of PI3K-AKT-mTOR pathway in breast cancer growth and metastasis, we use rapamycin, an inhibitor of mTOR protein, to suppress the PI3K-AKT-mTOR pathway. We found the downregulation of p-PI3K, p-AKT and mTOR being induced (Fig. 6c). All the above results confirmed that the combination of PGC-1β and FOXA2 inhibited breast cancer cells proliferation, migration and induced apoptosis through regulating the PI3K-AKT-mTOR signaling pathway.

## Discussion

It has been reported that the PGC-1 family exerts an important role in cancer metabolism and progression [35, 36]. PGC-1β is a member of this family and acts as a central regulator in energy metabolism and other biological processes. And it exerts an important role in different cancers: Kumazoe et al. proved the FOXO3/PGC-1β signaling axis was essential for sustain the pancreatic ductal adenocarcinoma cancer stem cell properties [37]; Bellafante et al. reported the overexpression of PGC-1β has the ability to provide balance between enhanced mitochondrial activity and promoted intestinal carcinogenesis [38]. However, the direct correlation and specific molecular mechanism between PGC-1β and breast cancer development remain unclear. Our previous research found that inhibition of endogenous PGC-1β induced cell apoptosis by regulating the mTOR pathway. In this study, we identified the PGC-1β was highly expressed in the progesterone receptor positive cell line MCF-7 and the high metastatic cell line MDA-MB-231. Meanwhile, PGC-1β was frequently overexpressed in breast cancer tissues compared to adjacent non-cancerous tissues. According to the clinical data, PGC-1β expression is strongly correlation with breast cancer lymph node metastasis or progesterone receptor expression, which was consistent with the expression characteristics in the two cell lines. These results demonstrated that PGC-1β may be involved in the occurrence and metastasis of breast cancer.

FOXA2 is a member of forkhead/winged-helix family, which plays crucial role in organ genesis, cellular metabolism, and tumor development [39]. FOXA2 has the function of promoting cell proliferation and cancer stem cell maintenance in triple-negative breast cancer [28]. In lung adenocarcinoma, the developmental transcription factors FOXA2 and Cdx2 function cooperatively with Nkx2-1 as an important regulator in inhibiting metastasis [40]. Previous research has demonstrated that the coactivation of FOXA2 through PGC-1β promotes fatty acid oxidation and triglyceride/VLDL secretion in the liver [41]. Based on the above studies, we hypothesized the PGC-1β may cooperate with FOXA2 to regulate the occurrence and development of breast cancer. As expected, we found PGC-1β and FOXA2 colocalized in the nucleus, and PGC-1β interference combined with FOXA2 overexpression significantly inhibited the proliferation and migration of breast cancer cells. These results indicated that PGC-1β and FOXA2 can physically interact with each other, and FOXA2 is essential for PGC-1β-induced biological function in breast cancer.

PI3K-AKT-mTOR is one of the major intracellular pathways, which serve a critical regulatory role among different kinds of tumor. mTOR is a key kinase of PI3K/AKT downstream, it could regulate tumor cell proliferation, growth, survival and angiogenesis [42–44]. mTOR protein being proved to be regulated by two pathways. Including PI3K-AKT-mTOR pathway and PDK1-ERK/MAPK-mTOR pathway [45]. Previous research proved that the PI3K-AKT-mTOR pathway involves the function of PGC-1β in regulating apoptosis of breast cancer cells. In this study, we detected the proteins expression of PI3K-AKT-mTOR signal pathway after transfected with PGC-1β and/or FOXA2 in breast cancer cells. The results showed PGC-1β cooperating with FOXA2 exerted their biological functions in breast cancer through regulating the PI3K-AKT-mTOR pathway. Although we have studied the impact of PGC-1β on breast cancer progression, there are still some deficiencies that need to be improved. More extensive research need to be explored to understand the mechanism of PGC-1β in breast cancer.

## Conclusions

In conclusion, our study proved that PGC-1β was high expression while FOXA2 was low expression in breast cancer tissues and cell lines. Downregulation of PGC-1β combined with overexpression of FOXA2 obviously inhibited the breast cancer cells proliferation, migration and induced apoptosis through regulating the PI3K-AKT-mTOR signaling pathway. Our findings reveal that PGC-1β may be serve as valuable diagnostic biomarker and potential therapeutic target for the development of breast cancer.

## Abbreviations

PGC-1β: peroxisome gamma coactivator-1β
FOXA2: forkhead box protein A2
qRT-PCR: real time quantitative reverse transcription-PCR
sh-PGC-1β: shRNA for PGC-1β
NC: shRNA-NC
PGC-1β: pcDNA3.1-PGC-1β
FOXA2: pcDNA3.1-FOXA2
Co-IP: co-immunoprecipitation
IHC: immunohistochemistry
IF: immunofluorescence
ANOVA: analysis of variance
